# The response of an egg parasitoid to substrate-borne semiochemicals is affected by previous experience

**DOI:** 10.1038/srep27098

**Published:** 2016-06-02

**Authors:** Ezio Peri, Gianandrea Salerno, Takoua Slimani, Francesca Frati, Eric Conti, Stefano Colazza, Antonino Cusumano

**Affiliations:** 1 Dipartimento di Scienze Agrarie e Forestali, Università degli Studi di Palermo, Viale delle Scienze, 90128, Palermo, Italy; 2 Dipartimento di Scienze Agrarie, Alimentari e Ambientali, Università degli Studi di Perugia, Borgo XX Giugno 74, 06121 Perugia, Italy; 3 Laboratory of Entomology, Wageningen University, Droevendaalsesteeg 1, 6708PB Wageningen, The Netherlands

## Abstract

Animals can adjust their behaviour according to previous experience gained during foraging. In parasitoids, experience plays a key role in host location, a hierarchical process in which air-borne and substrate-borne semiochemicals are used to find hosts. In nature, chemical traces deposited by herbivore hosts when walking on the plant are adsorbed by leaf surfaces and perceived as substrate-borne semiochemicals by parasitoids. Chemical traces left on cabbage leaves by adults of the harlequin bug (*Murgantia histrionica*) induce an innate arrestment response in the egg parasitoid *Trissolcus brochymenae* characterized by an intense searching behaviour on host-contaminated areas. Here we investigated whether the *T. brochymenae* response to host walking traces left on leaf surfaces is affected by previous experience in the context of parasitoid foraging behaviour. We found that: 1) an unrewarded experience (successive encounters with host-contaminated areas without successful oviposition) decreased the intensity of the parasitoid response; 2) a rewarded experience (successful oviposition) acted as a reinforcing stimulus; 3) the elapsed time between two consecutive unrewarded events affected the parasitoid response in a host-gender specific manner. The ecological role of these results to the host location process of egg parasitoids is discussed.

Learning and experience allow animals to adjust their behavioural responses to track changing ecological conditions. In insect parasitoids, whose eggs and larvae develop in or on the body of other arthropods, experience plays a key role when foraging in complex environments that undergo spatial and temporal variation in host resource availability^1^. The fitness of parasitoids strongly depends on their ability to find suitable hosts, as there is a direct link between host encounter rate and progeny production^2^. Insect parasitoids locate their hosts using a series of behavioural steps that are primarily mediated by chemical cues directly or indirectly related to the host^3^. At the initial steps of the host location process, many parasitoids innately respond to air-borne chemical cues (generally highly detectable but low reliable) whereas substrate-borne chemical cues (generally low detectable but highly reliable) become progressively more important after parasitoids have landed on a host-infested patch^4^,^5^,^6^. However, these instinctive behavioural responses displayed by parasitoids can be adjusted by successive experience in order to perform optimally in changing environments^7^,^8^,^9^,^10^. During the host location process, parasitoids can also learn to respond to novel cues (conditional stimuli) when found associated with key rewarding stimuli such as hosts or host-by products (unconditional stimuli)^1^. Through associative learning, wasps can exploit cues that are not reliable predictors of the host presence over the evolutionary time but that became associated with the host presence over the lifetime of the parasitoid^1^,^11^.

Investigations aimed at studying the parasitoid foraging behaviour in response to experience and learning have focused mainly on air-borne semiochemicals, whereas substrate-borne semiochemicals have received less attention^12^,^13^,^14^,^15^. In the case of parasitoids attacking herbivore hosts, it is important to consider the role played by the plant when studying experience and learning as some air-borne cues only elicit a response in a presence of a suitable background odour^16^. Wasps exploiting substrate-borne semiochemicals have to cope with potentially confounding leaf morphological features such as trichomes or veins when walking on the plant^17^,^18^,^19^. In addition, wasps must discriminate between components of the contact kairomone from similar components present in the plant cuticle, particularly chemical compounds such as linear and branched-hydrocarbons which can be present in both components^6^,^20^,^21^.

Previous studies showed that chemical traces left on cabbage leaves by adults of the harlequin bug *Murgantia histrionica* Hahn are perceived as kairomones by the egg parasitoid *Trissolcus brochymenae* Ashmead^22^. Once in contact with an area contaminated by adult traces, female wasps innately show an initial prolonged motionless period with the antennae kept in contact with the surface (an arrestment response), followed by an intense searching behaviour characterized by increased turning frequency (klinokinesis) and decreased walking speed (negative orthokinesis) (reviewed by^6^). The hierarchical value of the adult traces depends on the gender and on the physiological status of hosts; female parasitoids can discriminate between contact kairomones left by male and female bugs, spending more time on the patches contaminated by female bugs that have mated but not yet laid eggs^23^,^24^. Considering that host quality typically decreases as eggs develop, this fine-tuned foraging strategy based on indirect-host related contact cues is particularly adaptive because it maximizes the probability of *T. brochymenae* locating freshly laid *M. histrionica* eggs which are more suitable for parasitoid offspring^25^. However, how *T. brochymenae* females adjust their foraging behaviour in response to previous experience is not known, and little knowledge is available in general for egg parasitoids associated with heteropteran hosts. The few studies conducted so far demonstrated that the egg parasitoid *Trissolcus basalis* (Wollaston) changed the innate searching behaviour on *Nezara viridula* (L.) host traces according to previous experience gained during foraging^26^,^27^. The response on filter paper arenas contaminated with host traces becomes weaker when wasps re-encountered patches without oviposition rewards, whereas females respond strongly on kairomone patches where oviposition occurred^26^. The time window needed by *T. basalis* females to “forget” unrewarded experiences on female traces was estimated to be about 3 days, although it can be extended under stressful temperature regimes^27^. After forgetting an unrewarded experience, female wasps that encountered a contaminated filter paper arena behave similarly to naïve females^26^. However, these studies have not taken into account the role of the plant surface as the real interface for host-parasitoid interactions in nature.

In this study, we investigated the role of parasitoid experience on indirect-host related contact cues using the tritrophic system *Brassica oleracea* L. (Brassicaceae) – *M. histrionica* (Heteroptera: Pentatomidae) – *T. brochymenae* (Hymenoptera: Platygastridae). We studied how unrewarding (lack of oviposition) and rewarding experiences (successful oviposition) change the innate response of *T. brochymenae* towards host walking traces in the context of the parasitoids’ host location process. As parasitoids respond innately to host walking traces, the variable-response model proposed by^11^ is used to generate predictions rather than classical conditioning theory in which an unconditioned stimulus is paired with a conditioned stimulus^28^. The variable-response model aims to predict how and when learning modifies the parasitoid behaviour. According to the model, it is possible to hypothesize that female wasps that did not locate host eggs while exploring a leaf surface contaminated with host walking traces should decrease the intensity of their response to the same stimulus in the near future, whereas the response of female wasps that successfully located and parasitized host eggs should be reinforced. We also hypothesize that the response of female wasps should be affected by the elapsed time between two consecutive unrewarded events in a host-gender specific manner, due to the different hierarchical value of the adult traces.

## Results

### Experiment 1: unrewarded experience

**Figure 1:**
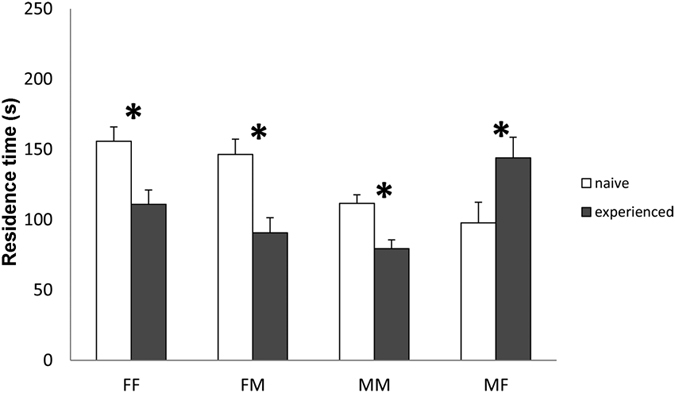
Behavioural response of *Trissolcus brochymenae* with unrewarded experience. Mean (±SE) residence time of *Trissolcus brochymenae* females encountering for the first time (naïve, white bars) or re-encountering (experienced, grey bars) *Murgantia histrionica* adult walking traces adsorbed on cabbage leaf disks. MM = experienced and tested on male traces, N = 31; MF = experienced on male traces and tested on female traces, N = 29; FM = experienced on female traces and tested on male traces, N = 27; FF = experienced and tested on female traces, N = 26. Asterisks indicate significantly different means within each pairwise combination (GLMM, *P < 0.05).

The residence time of *T. brochymenae* wasps was significantly affected by the treatment × experience interaction (χ^2^ = 7.46; df = 3; *P* < 0.001). Among each of the four treatments, experience status had a large and statistically significant effect on the wasps’ residence time (Fig. 1). Wasps experienced with host female chemical residues spent less time on the arena when re-encountering the same type of chemical cue (group FF: χ^2^ = 13.88; df = 1; *P* < 0.001). A similar effect was found for those parasitoids that were experienced on female walking traces and re-tested on male walking traces (group FM: χ^2^ = 16.24; df = 1; *P* < 0.001). Naïve parasitoids that were tested on host male traces displayed a reduced residence time when re-tested on the same type of chemical cue (group MM: χ^2^ = 13.31; df = 1; *P* < 0.001). On the contrary, wasps experienced on male traces and re-tested on female traces increased the time spent on the arena (group MF: χ^2^ = 11.90; df = 1; *P* < 0.001) (Fig. 1).

### Experiment 2: rewarded experience

**Figure 2:**
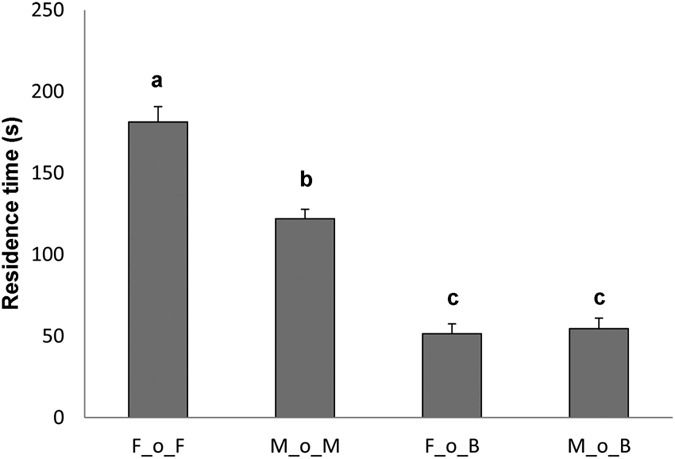
Behavioural response of *Trissolcus brochymenae* with rewarded experience. Mean (±SE) residence time of *Trissolcus brochymenae* females allowed to oviposit on a host egg mass in the presence of either host female or male traces and then tested according to different conditions: F_o_F = oviposition on female traces and tested on female traces, N = 32; M_o_M = oviposition on male traces and tested on male traces, N = 31; F_o_B = oviposition on female traces and tested on uncontaminated cabbage leaf disks, N = 35; M_o_B = oviposition on male traces and tested on uncontaminated cabbage leaf disks, N = 34. Different letters above bars indicate significantly different means (GLM, *P < 0.05).

The residence time of *T. brochymenae* wasps when tested in arenas contaminated by host chemical cues was significantly affected by previous successful oviposition experience (GLM, χ^2^ = 38.14; df = 3; *P* < 0.001) (Fig. 2). Rewarded parasitoids re-encountering host female residues (group F_o_F) showed longer arena residence time compared to rewarded parasitoids re-encountering host male residues (group M_o_M) (GLM, *z* = 2.83, *P* < 0.05). The influence of oviposition experience *per se* on residence time could be excluded because wasps with successful oviposition experience did not stay longer when re-tested on untreated arenas (Fig. 2). In fact, the arrestment response of rewarded wasps in the presence of host female residues was significantly different from controls (group F_o_B) (*z* = −7.56, P < 0.001). Similarly, the residence time of rewarded wasps on host male chemical cues was significantly different than controls (group M_o_B) GLM, *z* = −5.43, *P* < 0.001).

### Experiment 3: hierarchical value of host traces

**Figure 3:**
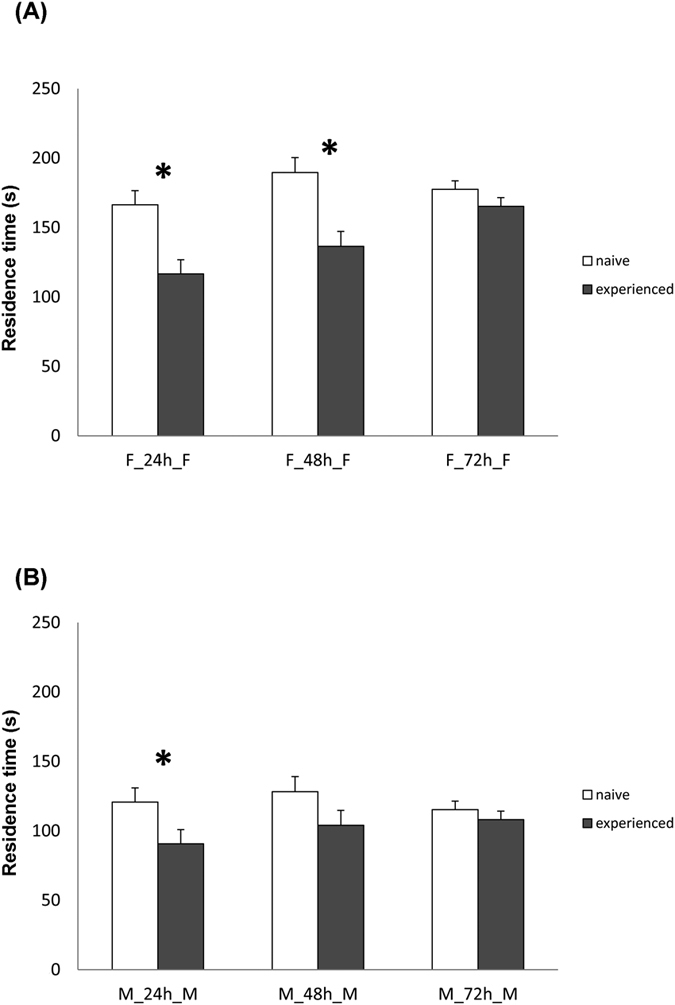
Behavioural response of *Trissolcus brochymenae* for evaluation of hierarchical value of host traces. Mean (±SE) residence time of *Trissolcus brochymenae* females trained and tested on 2 consecutive leaf disk arenas contaminated with traces of *Murgantia histrionica* adults (combination “female-female, F_F” in figure A or “male-male, M_M” in figure B) at intervals of 24, 48, or 72 h (experienced, grey bar), F_24 h_F, N = 30, F_48 h_F, N = 28, F_72 h_F, N = 29; M_24 h_M , N = 27, M_48 h_M, N = 28, M_72 h_M, N = 30. As controls, *T. brochymenae* females tested at the same times as experienced females were used (white bars; naïve females). Asterisks indicate significantly different means within each pairwise combination (GLM, *P < 0.05).

The residence time of experienced wasps was significantly affected by the host gender (χ^2^ = 5.42; df = 1; *P* < 0.01) and by the time interval (χ^2^ = 3.32; df = 2; *P* < 0.05) whereas the host gender × time interval interaction was not significant (χ^2^ = 0.15; df = 2; *P* = 0.705). The residence time of *T. brochymenae* wasps experienced on female traces and re-tested on female traces was significantly affected by the time interval × experience interaction (χ^2^ = 6.08; df = 2; *P* < 0.01) (Fig. 3a). Within each time interval treatment, the arena residence time was significantly different between naïve and experienced wasps as long as the time interval was shorter than 48 h (group F_24_F, χ^2^ = 9.46; df = 1; *P* < 0.01; group F_48_F, χ^2^ = 9.01; df = 1; *P* < 0.01; group F_72_F, χ^2^ = 0.27; df = 1; *P* = 0.601) (Fig. 3a). The residence time of *T. brochymenae* wasps was also significantly affected by time interval × experience interaction when parasitoids were experienced on male traces and re-tested on male traces (χ^2^ = 3.27; df = 2; *P* < 0.05) (Fig. 3b). However, within each time interval treatment, the arena residence time was significantly different between naïve and experienced wasps only when the time interval was not longer than 24 h (group M_24_M, χ^2^ = 8.46; df = 1; *P* < 0.01; group M_48_M, χ^2^ = 2.01; df = 1; *P* = 0.106; group M_72_M, χ^2^ = 0.55; df = 1; *P* = 0.462) (Fig. 3b).

## Discussion

In this work we demonstrated that *T. brochymenae* females adjust their innate behavioural response towards *M. histrionica* chemical traces on natural substrates according to prior experience. This suggests that experience is likely to play an important role in the host location process of egg parasitoids associated with heteropteran hosts after landing on an infested plant. Experience on host-related chemical cues may also have broader relevance, as many herbivores leave chemical residues when moving around on the plant, which can be perceived as host location kairomones by their natural enemies^20^,^21^,^29^,^30^.

Insect parasitoids exploiting substrate-borne chemical cues can adopt different searching patterns, depending on whether they receive either directional information about the location of the host (trail-following search) or information suggesting that a host is nearby, without giving a precise indication regarding its location (biased-random search)^3^,^6^. In the case of *T. brochymenae*, females adopt a biased-random search as consequence of an innate response induced by walking traces leaf by adults of *M. histrionica*^23^,^24^. Thus, once landing on an infested plant, the possibility of finding host eggs increases because of delayed flight tendency, prolonged stay on the plant, and extensive walking exploration. Our results suggest that the patch time allocation of *T. brochymenae* females could be modified by the reproductive success accumulated while foraging on plant surfaces contaminated with contact kairomones left by *M. histrionica* (Fig. 2). An oviposition reward increased the intensity of response of *T. brochymenae* to host female traces, a phenomenon that seems in agreement with the type of experience defined as “α-conditioning”^31^,^32^ in which rewards enhance innate responses towards a given stimulus. In contrast, *T. brochymenae* females displayed a decrease in the intensity of their behavioural responses when unrewarded individuals were re-tested on leaf disks contaminated with the same type of indirect host-related cues (Fig. 1). In studies testing associative learning, unrewarding experiences induce wasps to cease responding to the learned cues^33^,^34^. In the parasitoid *Microplitis croceipes* Cresson, a negative experience (i.e. oviposition in an unsuitable host) significantly decreases the learned response to vanilla induced by antennating host frass with the odour^35^. For *T. brochymenae*, the adaptive significance of unrewarded experience may be that chemical traces left by *M. histrionica* adults cannot guarantee the presence of host eggs or provide directional information^4^. Hence, even in patches contaminated by ‘promising host cues’, it could be adaptive for parasitoids to lose their searching motivation and leave the area if hosts are not found after a certain amount of time^4^. In fact, in insect parasitoids, patch leaving tendency is assumed to increase over time but it can be adjusted by successful oviposition events^36^,^37^,^38^,^39^,^40^. Rewarded and unrewarded experiences in *T. brochymenae* affect the subsequent level of response towards host traces in agreement with the prediction that the response potential of a stimulus can change as consequence of learning^11^.

Our results show that over a period of some days, the behavioural response of *T. brochymenae* was influenced by the time elapsed between two consecutive unrewarded events. As expected, residence time of wasps with a previous unrewarded experience tended to increase relative to naïve controls, suggesting that parasitoids “forgot” an encounter with host walking traces adsorbed on leaf disks without successfully locating a host egg mass. Interestingly we found a host gender-specific effect when comparing the results of the residence time between naïve and experienced parasitoids as wasps’ behavioural responses differed over time depending on whether experience occurred on male- or female host traces (Fig. 3). A first possible hypothesis to explain our results is that parasitoids forgot unrewarded experiences on male-associated cues faster than experiences with female-associated cues. This appears to support the theory that the temporal window of previous experience is affected by the rank of how well-correlated stimuli are with their associated resources^1^,^41^, since female host traces, which should be more strongly associated with host egg presence, elicit a more intense response. A second possible explanation is that the host-gender effect shown in our experiments may be simply due to differences in strength of reaction between parasitoid responses to female and male host traces as different stimuli used in the foraging process can elicit different levels of response^11^. Under such hypothesis, the group of parasitoids tested on male traces may fail to reach significance when the time interval was longer than 24 h due to a weaker level of response, and not due to a higher tendency to forget. In fact, an aspect that limits our current understanding of the results is the inability to link the behavioural responses adjusted by experience with the memory structure possessed by *T. brochymenae*. Depending on the variability of the environment and the reliability of host-related cues, insect parasitoids have been shown to retain information in different memory phases, e.g. short-term memory (STM), medium-term memory (MTM) or long-term memory (LTM), which differ in terms of stability, duration and energy consumption^1^,^42^,^43^,^44^,^45^. To discriminate between the two hypotheses, further studies should investigate whether *T. brochymenae* females use different forms of memory to store previous foraging experiences on traces left by male and female *M. histrionica* hosts even if our results may suggest that unrewarded experience is stored in medium-term memory.

The role of experience on substrate-borne chemical cues has been previously investigated in the egg parasitoid *T. basalis* when searching for *N. viridula* host eggs^26^,^27^. Overall, strong similarities were found between *T. basalis* and *T. brochymenae* as in both species the behavioural response decreases after one hour in unrewarded females and increases in rewarded females. However, while the innate *T. basalis* response to host male traces was not modified by experience gained during foraging, an opposite outcome was found for *T. brochymenae* suggesting that experience is more relevant for the latter species. It is possible that such differences can be related to the oviposition strategies evolved by the herbivore species, which consequentially affect the spatial distribution of the host resources for the associated egg parasitoids. While the oviposition strategy of *N. viridula* (the main host of *T. basalis*) consists of laying 2–3 egg masses each made up of about 100–120 eggs, *M. histrionica* (the main host of *T. brochymenae*) produces about 8–9 egg masses which consist only of up to 12 eggs each^46^,^47^. Consequently, a *T. brochymenae* female should be expected to encounter a higher number of egg masses during their lifetime than *T. basalis,* and so a finely-tuned response to experience could be particularly adaptive in the former species. In fact, it has been suggested that the number of lifetime learning events can influence the value of experience, leading to the prediction that when hosts are encountered once or only a few times, experience is less likely to be relevant^48^. In fact, in the parasitoid *Melittobia digitata* Dahms, the innate response to contact kairomones was not affected by previous host exposure experience^49^. This could be because *M. digitata* females usually locate and parasitize only a single host and thus, prior host experience would not be favoured by selection in this parasitoid species. Alternatively, the differences found between *T. brochymenae* and *T. basalis* could be explained by a difference in the chemistry of walking traces between females and males of their respective hosts, under the hypothesis that this difference is larger between females and males in *N. viridula* (the host of *T. basalis*) as compared to *M. histrionica* (the host of *T. brochymenae*). In the case of *T. basalis*, host gender discrimination is based on *n*-nonadecane, a cuticular hydrocarbon that is present only in walking traces of *N. viridula* males but not on those left by females^50^ whereas the chemical nature of host gender discrimination by *T. brochymenae* is still unknown. To test this alternative hypothesis, further studies are required to identify the chemical differences between walking traces of males and females of *M. histrionica* in order to clarify which compound(s) is involved in host sex discrimination by *T. brochymenae*. Finally, it is not possible to exclude that methodological differences in the substrate used in the two studies (filter paper arenas for *T. basalis* and leaf disks for *T. brochymenae*) can be responsible for the different outcome found in terms of experience on male host traces. However, as detection of host kairomones should be facilitated in simplified experimental conditions in which parasitoids do not have to cope with potentially confounding leaf features, it is unlikely that the innate *T. basalis* response to host male traces would be modified by previous experience even if more natural substrates would have been employed.

In conclusion, our results contribute to a better understanding of the role played by experience in the behavioural response to indirect host-related cues by egg parasitoids. We show that parasitoid experience can be expressed in a host gender-specific manner, and that this phenomenon is observed in a tritrophic context where natural substrates are present.

## Methods

### Insect colonies

Harlequin bugs (*M. histrionica*) were originally collected from cabbage in Beltsville, USA in 2000 whereas *Trissolcus brochymenae* adults were obtained from *M. histrionica* eggs found in San Diego, USA in 2000. Both insects were maintained in quarantine conditions in a growth chamber (25 ± 1 °C, 60 ± 5% RH, 16 h:8 h light:dark) at the Entomology laboratories of the University of Perugia, Italy. The colony of *M. histrionica* was reared in plastic cages and fed with cabbage leaves. Every 2–3 days, last instar nymphs were individually isolated into a single plastic pot (Ø = 40 mm, height = 65 mm) and observed daily until adult emergence, so that individuals of known ages were continuously available. Bugs used for bioassay were mated adult males and females approximately 10–14 days postemergence, with females in pre-ovipositional state. Mated adults were obtained from pairs separated after mating and isolated individually for 24 h before experiments.

The *T. brochymenae* colony was reared on *M. histrionica* eggs, and adult wasps were kept in 85 ml glass tubes provided with honey-water solution. After emergence, male and female parasitoids were kept together to allow mating. In all of the experiments, female wasps were 2–3 days old, mated and naïve (i.e., they had not previously encountered adult host chemical cues). Female wasps were individually isolated in small vials 24 h before bioassays and were allowed to acclimatize to the conditions of the bioassay room for at least 30 min before testing.

### Plants

*Brassica oleracea* plants (var Italica cv Marathon) were grown under controlled greenhouse conditions (25 ± 3 °C, 50 ± 10% relative humidity) from seeds in polystyrene pots filled with peat. After one week, seedlings were transplanted individually in 14 cm diameter plastic pots, fertilized with commercial soil (Trflor—HOCHMOOR), and watered as needed. All experiments were carried out using 5–6 week old plants.

### General bioassay procedure

To assess parasitoids’ response to host walking traces adsorbed on the leaf surface, we used a protocol similar to that of 22. Briefly, bioassays were conducted in an open arena consisting of the adaxial surface of a leaf disk (5 cm in diameter) exposed to a single female or male bug for 30 min to contaminate it with the stink bug’s walking traces. Adults with excised stylets were used as described by^22^ in order to prevent bugs from feeding and thus to obtain leaf disks contaminated only with chemical traces. A single parasitoid female was then released into the centre of the leaf and observed until the wasp flew away or reached the leaf disk borders. The residence time of the wasp spent on the leaf disk, which in several past studies is strongly correlated with increased turning frequency and slower walking speed^23^,^26^,^27^, was recorded as a measure of the intensity of each parasitoid’s response towards host traces. The residence time of naïve wasps on untreated leaf disks is about 5–10 seconds (Cusumano, personal observation). Experiments were conducted between 9:00 to 13:00 in an isolated room maintained at 25 ± 1 °C, with lighting on the arena provided by two 19 cm long fluorescent tubes.

### Experiment 1: unrewarded experience

The aim of this first experiment was to examine the behavioural response of wasps encountering successive leaf areas contaminated with male or female host traces without successful oviposition (unrewarding experience). Naïve wasps were first tested on host-contaminated areas. After displaying the typical arrestment behaviour and leaving the patch, these wasps were considered experienced. They were then recaptured, isolated in small vials and re-tested 1 h later on newly treated arenas according to the four following treatments: (1) naïve and experienced wasps were both tested on female traces (Female-Female, FF), (2) naïve wasps were tested on female traces but experienced wasps were tested on male traces (Female-Male, FM), (3) naïve and experienced wasps were both tested on male traces (Male-Male, MM), or (4) naïve wasps were tested on male traces but experienced wasps were tested on female traces (Male-Females, MF). For each treatment 26–31 successful replicates were performed.

### Experiment 2: rewarded experience

In a second experiment, the influence of a successful (i.e., rewarded) oviposition experience on the wasps’ behavioural responses was investigated. Single, naïve *T. brochymenae* wasps were released onto a leaf disk contaminated with residues of host males or females and with a host egg mass (five eggs) in the middle of the leaf disk. During this training phase, the residence time of the wasp was not recorded. Then, experienced wasps (i.e. those that had located the egg mass and parasitized one egg) were recaptured and kept isolated in a small vial for 1·h. They were then tested on cabbage leaf surfaces treated with chemical residues from host females or males as previously described, and their residence time was recorded. These tests were run using two treatment combinations: (1) oviposition on female traces and tested on female traces (Female_oviposition _Female, F_o_F) or (2) oviposition on male traces and tested on male traces (Male_oviposition_Male, M_o_M). For egg parasitoids of stink bugs, it is known that a successful oviposition generally increases turning frequency, decreases walking speed and increase residence time in a manner analogous to contact with host chemical residues^51^. Thus, to separate the effect of oviposition experience and host chemical residues, experienced wasps tested on uncontaminated leaf disks were used as controls, leading to two additional treatments: (3) oviposition on female residues and tested on uncontaminated arena (Female_oviposition_Blank leaf, F_o_B), or (4) oviposition on male residues and tested on uncontaminated arena (Male_oviposition_Blank leaf, M_o_B). For each treatment 31–35 successful replicates were performed.

### Experiment 3: hierarchical value of host traces

A third experiment was carried out to evaluate the possible hierarchical value of areas contaminated by chemical residues left by host females or males on the parasitoids’ behavioural response, as a function of the elapsed time between two consecutive unrewarded events. Naïve wasps were first exposed to a leaf disk area treated with female or male walking traces of *M. histrionica*. During this training phase, the residence time of the wasp was not recorded. Then, experienced wasps (i.e., those that have left the patch after showing an arrestment response) were captured and kept in a small vial and fed with a drop of honey-water solution for 24, 48 or 72 h. These experienced wasps were then re-tested on another leaf disk treated with female or male walking traces. These tests were run using two host gender combinations: (1) testing naïve wasps on female traces and re-testing them on female traces after 24, 48 or 72 hours (F_24 h_F, F_48 h_F, F_72 h_F); (2) testing naïve wasps on male traces and re-testing them on male traces after 24, 48 or 72 hours (M_24 h_M, M_48 h_M, M_72 h_M). It has been demonstrated in several parasitoid species that extended periods without host contact can affect their host-searching behaviour (for a review, see^52^). Therefore, to disentangle the effect of unrewarded experience and increased duration without successful oviposition on wasps’ behavioural response, as a control we used naïve wasps kept in a vial for the same amount of time as experienced wasps. Control wasps were tested alternately with experienced females. For each treatment 27–30 successful replicates were performed.

### Statistical analyses

For the Experiment 1, we used a Generalized Linear Mixed Model (GLMM) with treatment combination, experience status, and treatment × experience interaction as fixed terms and parasitoid identity as a random term, with parasitoid residence time as the response variable. To further investigate the role of the experience status, we tested its effect in each pairwise treatment separately when a significant interaction was found. For Experiment 2, we fitted a Generalized Linear Model (GLM), testing the dependence of parasitoid residence time on oviposition treatment. If the model detected significant differences amongst factor levels, we proceeded to pairwise comparisons to determine which differed using the *glht* function found in the *multcomp* package of the R software^53^. For experiment 3 we first fitted a GLM with time interval, host gender and the time interval × host gender interaction as explanatory factors using parasitoid residence time of experienced wasps as response variable. We then analysed the data separately for each of the two combinations (Female-Female, Male-Male) as a significant effect of host gender was found (see results) and we were interested in the dependence of parasitoid response on the host gender over time. Thus for each combination, we fitted a GLM with time interval, experience status, and the time interval × experience interaction as explanatory factors and parasitoid residence time as the response variable. To further investigate the role of the experience status, we tested its effect in each time interval treatment (24, 48 or 72 hours) when a significant time interval × experience interaction was found. For all of the GLMM and GLM analyses above, we assumed a gamma error distribution with a reciprocal link function, since residence time data were not normally distributed (typical for time-to-event data), and the variance changed faster than linearly with the mean. Significance of the fixed terms in the model was determined using Likelihood Ratio Tests (LRTs) comparing the full model with and without the factor in question^54^. Model fit was assessed with residual plots. All statistical analyses were performed with R software version 3.1.3^55^.

## Additional Information

**How to cite this article**: Peri, E. *et al.* The response of an egg parasitoid to substrate-borne semiochemicals is affected by previous experience. *Sci. Rep.* **6**, 27098; doi: 10.1038/srep27098 (2016).
